# 顶空进样-双柱双检测器-气相色谱法分析人体血液中乙醇等12种挥发性化合物

**DOI:** 10.3724/SP.J.1123.2023.12015

**Published:** 2024-09-08

**Authors:** Qiongying ZHENG, Yujie ZHI, Wenjia DUAN, Min LÜ, Yue XIAO, Ping XIANG, Hang CHEN, Keming YUN

**Affiliations:** 1.山西医科大学法医学院,山西晋中 030600; 1. College of Forensic Medicine, Shanxi Medical University, Jinzhong 030600, China; 2.司法鉴定科学研究院,司法部司法鉴定重点实验室, 上海市法医学重点实验室,上海市司法鉴定专业技术服务平台,上海市标准化创新中心(司法鉴定),上海 200063; 2. Academy of Forensic Science, Key Laboratory of Forensic Science, Ministry of Justice, Shanghai Key Laboratory of Forensic Medicine, Shanghai Forensic Service Platform, Shanghai Innovation Center of Standardization (Forensic Medicine), Shanghai 200063, China; 3.中国药科大学药学院,江苏南京 210009; 3. School of Pharmacy, China Pharmaceutical University, Nanjing 210009, China

**Keywords:** 顶空进样, 双柱双检测器, 气相色谱法, 挥发性化合物, 人体血液, 标准化, headspace injection (HS), double-column dual-detector, gas chromatography (GC), volatile compounds, human blood, standardization

## Abstract

基于GB/T 42430-2023与GA/T 204-2019的技术方法,分别在两套不同的设备平台及色谱柱体系上建立了同时分析人体血液中乙醇等12种挥发性化合物的顶空进样-双柱双检测器-气相色谱分析方法。所建立方法中酮醇类、苯类物质色谱分离基本良好;乙醇等7种酮醇类化合物线性范围为0.10~3.00 g/L,线性相关系数(*r*)均大于0.997,检出限除正丙醇为0.005 g/L外,其余化合物均为0.05 g/L,定量限均为0.10 g/L,符合标准要求;苯等5种苯类化合物的线性范围为0.05~50 mg/L,*r*均大于0.995,检出限均为0.02 mg/L,定量限均为0.05 mg/L,符合标准要求;色谱柱J&W DB-BAC1 UI和色谱柱Rtx-BAC-PLUS 2上各化合物不同加标水平下的平均回收率为92.2%~111.6%,相对标准偏差(*n*=6)为0.4%~7.4%。对两套不同的设备平台及色谱柱体系上乙醇的测定进行不确定度评定,乙醇的不确定度主要来源于校准曲线,取置信概率95%(包含因子*k*=2),能力验证样品1在色谱柱J&W DB-BAC1 UI和色谱柱Rtx-BAC-PLUS 2的测定结果分别为(1.20±0.06) g/L和(1.20±0.07) g/L,样品2在色谱柱J&W DB-BAC1 UI和色谱柱Rtx-BAC-PLUS 2的测定结果分别为(0.78±0.04) g/L和(0.79±0.05) g/L。能力验证样本分析与*z*比分数评价展示了所建立方法的数据可靠性;甲醇及有机溶剂中毒患者样本分析结果表明,所建立方法适用于人体血液等体液中乙醇等7种酮醇类化合物和5种苯类化合物的高精度定量分析,可用于司法鉴定、中毒检测等实践场景。

## Abstract (en)

Based on the technical methods of GB/T 42430-2023 and GA/T 204-2019, this study established an analytical method for headspace injection double-column dual-detector (hydrogen flame ion detector) gas chromatography for the simultaneous analysis of at least 12 volatile compounds, including ethanol, in human blood using two different equipment platforms and chromatographic columns. A 100 μL blood or urine sample and a 0.04 g/L *tert*-butanol working solution prepared as an internal standard are introduced into the headspace sample bottle and then sealed, mixed, and placed on the headspace sampler rack. Using different equipment platforms and columns, methodological parameters such as the limit of detection (LOD), limit of quantification (LOQ), precision, and accuracy of the method were systematically evaluated. The chromatographic separation of acetone, alcohols and benzenes using the established method was satisfactory. The linear ranges, linear correlation coefficients (*r*), and LODs of acetone and six alcohols, including ethanol, were 0.10-3.00 g/L, >0.997, and 0.05 g/L, respectively. The LOQs were 0.10 g/L for all other compounds, excluding *n*-propanol (0.005 g/L). Additionally, the linear ranges, *r* values, LODs, and LOQs of benzene and four benzene derivatives were 0.05-50 mg/L, >0.995, 0.02 mg/L, and 0.05 mg/L, respectively (Column J&W DB-BAC1 UI and Column Rtx-BAC-PLUS 2). The average recoveries of compounds on J&W DB-BAC1 UI and Rtx-BAC-PLUS 2 columns ranged from 92.2% to 111.6%, and the relative standard deviations (RSDs, *n*=6) ranged from 0.4% to 7.4%. The LOD, LOQ, precision, accuracy, and linearity of the established method met the requirements of relevant standards, and no significant differences arose between the methodological parameters of the two platforms. CNAS-GL006 (2019) and JJF 1059.1-2012 were used as guides to evaluate the uncertainty of ethanol on two different sets of equipment platforms and chromatographic columns. The ethanol uncertainty was mainly derived from the calibration curve; however, the confidence probability was 95% (*k*=2). According to the analysis of the verification samples and real samples, the established method is suitable for the high-precision quantitative analysis of acetone and six alcohols and five benzene derivatives in human blood and other body fluids. It can be used in practical scenarios such as judicial identification and the detection of poisons.

自2020年起,醉酒驾车已成为全国法院审结刑事案件中数量占比最多的犯罪行为^[1⇓⇓-4]^。血液中乙醇含量作为罪与非罪的关键性科学证据,直接关系到裁判公正与社会治理。2023年8月6日,国家标准GB/T 42430-2023《血液、尿液中乙醇、甲醇、正丙醇、丙酮、异丙醇和正丁醇检验》^[5]^正式发布。

GB/T 42430-2023标准明确了“双柱双检测器”的刚性要求。依据国家标准进行技术分析和实验验证,形成可供实践的技术方法成为相关实验室目前紧急与必要的任务之一^[6]^。同时,其他挥发性物质如甲醇、苯、甲苯等小分子化合物的检测需求在法医学实践、临床检验、医疗急救、工业生产、科学研究等场景也较常见^[7⇓⇓⇓⇓-12]^。按照GB/T 42430-2023标准要求建立定量更加精准的分析方法有助于相关实践工作的精细化和细致化^[13]^。

为了解不同设备平台、不同色谱柱体系对GB/T 42430-2023的适应性,评价依据GB/T 42430-2023标准建立可拓展技术方法的可行性,本研究综合GB/T 42430-2023和公共安全行业标准GA/T 204-2019《法庭科学血液、尿液中苯、甲苯、乙苯和二甲苯检验 顶空气相色谱法》^[14]^,分别在两套不同的设备平台及色谱柱体系上建立了可在人体血液中至少同时分析乙醇等12种挥发性化合物的顶空进样-双柱双检测器-气相色谱应用分析方法。依据上述标准及司法行政行业标准SF/T 0063-2020《法医毒物分析方法验证通则》^[15]^要求,分别对所建立方法进行完整的方法学验证,并依照标准进行评价,通过实际样本测试,对所建立方法的实效性进行初步评价。同时,根据中国合格评定国家委员会发布的《化学分析中不确定度的评估指南》(CNAS-GL006: 2019)及国家质量监督检验检疫总局发布的《测量不确定度评定与表示》(JJF 1059.1-2012)对两套不同的设备平台及色谱柱体系上乙醇的测定进行了不确定度评定,以乙醇的不确定度评定为例,通过对乙醇不确定度各分量分析和结果评定,找出影响测定结果不确定度的主要因素及关键控制点。

## 1 实验部分

### 1.1 仪器、试剂与材料

7697A顶空进样器和8890气相色谱仪(安捷伦科技(上海)有限公司)。HS-12顶空进样器(北京中惠普分析技术研究所), GC 2000气相色谱仪(杭州谱育科技发展有限公司)。活塞代排式移液器(M100E(10~100 μL)、M250E(50~250 μL)及M1000E(100~1000 μL),美国Gilson公司)。SQP型电子天平(检定分度值(*e*)为1 mg,最小分度值(*d*)为0.01 mg,赛多利斯科学仪器(北京)有限公司)。钳口密封瓶(10 mL)、单面聚四氟乙烯(PTFE)硅胶垫片和钳口密封铝盖(上海雷布斯生物科技有限公司)。

甲醇购自德国Sigma Aldrich,纯度>99.9%;乙醇、甲苯购自上海凌峰化学试剂有限公司;异丙醇、正丙醇、苯购自上海安谱实验科技股份有限公司;异丁醇、正丁醇购自上海易恩化学技术有限公司;对二甲苯、邻二甲苯、间二甲苯、二甲亚砜购自国药集团化学试剂有限公司;叔丁醇购自美国ChemService公司;邻二甲苯纯度为98%,其余试剂纯度均>99.5%。实验用水均为Milli-Q超纯水系统(美国Millipore公司)制备的超纯水。

### 1.2 溶液配制

#### 1.2.1 系列混合校准工作溶液配制

根据GB/T 42430-2023和GA/T 204-2019中所推荐12种化合物的线性浓度,称取甲醇100.00 mg和其他6种酮醇类化合物各100.50 mg,用水定容至10 mL,配制成10.0 g/L的7种酮醇类化合物混合储备溶液Ⅰ;称取邻二甲苯10.20 mg和其他4种苯类化合物各10.05 mg,用二甲亚砜定容至10 mL,配制成1.0 g/L的5种苯类化合物混合储备溶液Ⅱ。

移取300 μL混合储备溶液Ⅰ、 500 μL混合储备溶液Ⅱ和200 μL水-二甲亚砜(1∶1, v/v)混合溶液配制成3.00 g/L的酮醇类化合物和500 mg/L的苯类化合物的混合工作溶液Ⅲ,并用水-二甲亚砜(1∶1, v/v)混合溶液稀释混合工作溶液Ⅲ得到系列混合校准工作溶液(酮醇类化合物的质量浓度依次为0.10、0.20、0.50、0.80、1.00、2.00、3.00 g/L,苯类化合物的质量浓度依次为0.50、1.00、2.00、5.00、10.00、25.00、50.00 mg/L)。

#### 1.2.2 叔丁醇内标溶液配制

根据叔丁醇标准物质的纯度,称取50.10 mg的叔丁醇标准物质,用水定容至10 mL,配制成5.0 g/L的叔丁醇储备溶液,移取5.0 g/L叔丁醇储备溶液适量,用水稀释,配制质量浓度为0.04 g/L的叔丁醇内标工作溶液,置于4 ℃冰箱中冷藏保存。

### 1.3 试样处理

取样品(血液或尿液)100 μL及叔丁醇内标工作溶液500 μL,置于钳口密封瓶内,盖上硅胶垫片,用密封钳加封铝帽,混匀,置于顶空进样器样品架上进样。

### 1.4 分析条件

#### 1.4.1 平台a

该仪器平台由7697A顶空进样器串联8890气相色谱仪组成,其中气相色谱仪同时装备两根色谱柱与两个氢火焰离子化检测器(FID)。顶空进样器设置参数如下:加热箱温度65 ℃;定量环温度105 ℃;传输线温度110 ℃;气相循环时间20 min;钳口密封瓶加热平衡时间10.0 min;钳口密封瓶加压时间0.10 min;定量环充满时间0.1 min;定量环平衡时间0.05 min;进样时间1.00 min。顶空进样器进样针连接气相色谱仪进样口,进样口后端通过传输管(去活熔融石英,内径0.53 mm)连接至微流路板无吹扫分流器,分流器两个输出端分别连接色谱柱a-Ⅰ(J&W DB-BAC1 UI, 30 m×0.32 mm×1.8 μm,美国Agilent公司)与色谱柱a-Ⅱ(J&W DB-BAC2 UI, 30 m×0.32 mm×1.2 μm,美国Agilent公司);两根色谱柱分别连接独立的FID。GC柱升温程序如下:40 ℃保持3 min,以10 ℃/min速率升至150 ℃,保持1 min。进样口温度150 ℃; FID温度250 ℃;氢气流量30 mL/min;空气流量400 mL/min;载气为氮气;柱流量4.0 mL/min。

#### 1.4.2 平台b

该仪器平台由HS-12顶空进样器串联GC 2000气相色谱仪组成,其中GC 2000气相色谱仪同时装备两根色谱柱与两个FID。HS-12顶空进样器参数同平台a,与GC 2000气相色谱仪链接方式同平台a,色谱柱b-Ⅰ(Rtx-BAC-PLUS 1, 30 m×0.32 mm×1.8 μm,美国Restek公司)与色谱柱b-Ⅱ(Rtx-BAC-PLUS 2, 30 m×0.32 mm×0.6 μm,美国Restek公司)的升温程序、进样口温度、检测器温度、空气流量、载气、主流量同平台a,氢气流量为40 mL/min。

## 2 结果与讨论

### 2.1 方法学验证

#### 2.1.1 专属性

平台a典型色谱图见图1a;相较其上一代产品J&W DB-ALC1&2色谱柱^[16]^,双柱均实现了对叔丁醇和丙酮的分离,但柱a-Ⅱ无法完全分离苯和异丁醇。这一结果展示了双柱双检测器系统对专属性的有益贡献。在缺乏其他分子识别手段(如质谱)的情况下,双柱、双检测器同时分析,能降低因干扰性共流出物导致的假阳性风险^[17]^。因此,GB/T 42430-2023明确了“双柱双检测器”的刚性要求,明确提出了以“双柱定性、单柱定量”,即两根性质不同的色谱柱结果进行定性,分离度良好的色谱柱进行定量为基本准则的技术要求。

**图1:**
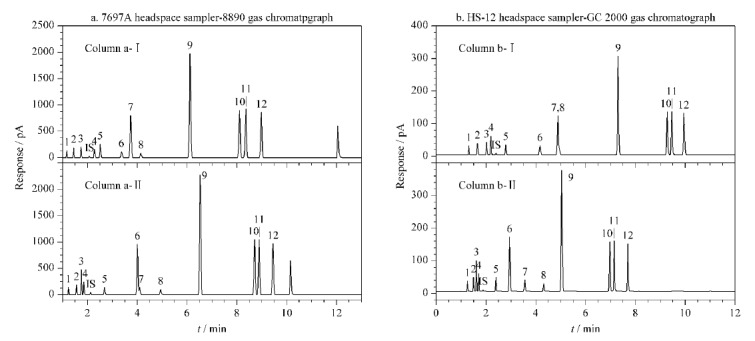
12种化合物及内标叔丁醇在(a)平台a和(b)平台b上的典型色谱图

平台b典型色谱图见图1b;其中柱b-Ⅱ对乙醇等12种化合物及内标叔丁醇分离良好,柱b-Ⅰ无法完全分离苯和正丁醇。柱b-Ⅰ、b-Ⅱ组合与柱a-Ⅰ、a-Ⅱ组合的典型色谱图略有区别,可能与其填料相关。

因柱a-Ⅰ、柱b-Ⅱ在本研究中分别对所分析的12种目标物及内标分离良好,在定量分析中,以柱a-Ⅰ、柱b-Ⅱ的测试结果分别作为平台a和平台b的定量数据来源。

#### 2.1.2 线性范围、检出限和定量限

取配制好的系列混合校准工作溶液,按照1.4节分析条件测定,分别选用柱a-Ⅰ、柱b-Ⅱ对系列混合溶液建立参考校准曲线。用各化合物的浓度值为横坐标,各化合物与叔丁醇内标的峰面积比为纵坐标,分别获得柱a-Ⅰ及柱b-Ⅱ对12种化合物的参考校准曲线(见表1)。各酮醇类化合物在不同条件下的线性相关系数(*r*)均大于0.997,符合GB/T 42430-2023的附录A.2要求,各苯类化合物在不同条件下的*r*值均大于0.995,符合GA/T 204-2019的附录A.1要求。各化合物均未见延迟效应。

**表1:** 12种化合物在柱a-Ⅰ和柱b-Ⅱ上的线性范围、相关系数、检出限和定量限

Column	Compound	Linear range	Linear equation	*r*	LOD	LOQ
a-Ⅰ	methanol	0.10-3.00	*y*=0.6794*x*-0.0399	0.9992	0.05	0.10
	ethanol	0.10-3.00	*y*=0.9193*x*-0.0762	0.9990	0.05	0.10
	isopropanol	0.10-3.00	*y*=1.5593*x*-0.127	0.9992	0.05	0.10
	acetone	0.10-3.00	*y*=2.8565*x*-0.1656	0.9995	0.05	0.10
	*N*-propanol	0.10-3.00	*y*=2.2476*x*-0.1746	0.9986	0.005	0.10
	isobutanol	0.10-3.00	*y*=2.4088*x*-0.196	0.9992	0.05	0.10
	*N*-butanol	0.10-3.00	*y*=2.0575*x*-0.0503	0.9994	0.05	0.10
	benzene	0.05-50	*y*=1.1112*x*-3.5888	0.9991	0.02	0.05
	toluene	0.05-50	*y*=0.7995*x*-3.0549	0.9984	0.02	0.05
	*p*-xylene	0.05-50	*y*=1.121*x*-3.9122	0.9991	0.02	0.05
	*m*-xylene	0.05-50	*y*=1.0954*x*-3.7899	0.9990	0.02	0.05
	*o*-xylene	0.05-50	*y*=0.7076*x*-3.4284	0.9987	0.02	0.05
b-Ⅱ	methanol	0.10-3.00	*y*=0.7166*x*-0.0573	0.9986	0.05	0.10
	ethanol	0.10-3.00	*y*=1.4533*x*-0.1259	0.9988	0.05	0.10
	isopropanol	0.10-3.00	*y*=3.7507*x*-0.2053	0.9994	0.05	0.10
	acetone	0.10-3.00	*y*=3.8073*x*-0.2351	0.9992	0.05	0.10
	*N*-propanol	0.10-3.00	*y*=2.6454*x*-0.2184	0.9994	0.005	0.10
	isobutanol	0.10-3.00	*y*=4.2931*x*-0.4383	0.9989	0.05	0.10
	*N*-butanol	0.10-3.00	*y*=2.9704*x*-0.2727	0.9991	0.05	0.10
	benzene	0.05-50	*y*=0.9121*x*-3.6015	0.9989	0.02	0.05
	toluene	0.05-50	*y*=1.1417*x*-4.0097	0.9986	0.02	0.05
	*p*-xylene	0.05-50	*y*=0.7722*x*-3.6543	0.9988	0.02	0.05
	*m*-xylene	0.05-50	*y*=0.9910*x*-3.4859	0.9986	0.02	0.05
	*o*-xylene	0.05-50	*y*=1.1091*x*-4.3205	0.9979	0.02	0.05

*y*: the ratio of the peak areas of the target to the internal standard *tert*-butanol; *x*: mass concentration. The units of linear ranges, mass concentrations, LODs and LOQs for acetone and alcohols are g/L. The units of linear ranges, LODs and LOQs for benzene compounds are mg/L.

以信噪比(*S/N*)分别≥3和≥10时的质量浓度作为方法的检出限(LOD)和定量限(LOQ),正丙醇的LOD为0.005 g/L,其余酮醇类化合物的LOD均为0.05 g/L;苯类化合物的LOD均为0.20 mg/L;酮醇类化合物的LOQ均为0.10 g/L,苯类化合物的LOQ均为0.50 mg/L。与GB/T 42430-2023和GA/T 204-2019相比,本研究建立方法的LOD和LOQ均满足现有标准要求。12种化合物在柱a-Ⅰ、柱b-Ⅱ上的线性范围、*r*、LOD、LOQ分别见表1。

对柱a-Ⅰ及柱b-Ⅱ获得的参考标准曲线进行协方差分析,各化合物参考标准曲线斜率的*P*值为0.46,截距的*P*值为0.30,均>0.05,说明两个仪器平台参考标准曲线不具有统计学差异。

#### 2.1.3 准确度与精密度

在空白血液中分别添加12种化合物的混合溶液(酮醇类各化合物质量浓度分别为0.20、0.80、2.00、3.00 g/L,苯类各化合物质量浓度分别为0.50、1.00、5.00、25.00 mg/L)进行加标回收试验,每个加标水平连续测定6次,计算回收率和相对标准偏差(RSD)。平台a各化合物的回收率为92.2%~111.6%, RSD为0.4%~7.4%(*n*=6,表2),平台b各化合物的回收率为94.2%~111.2%, RSD为0.4%~4.7%(*n*=6,表2),表明该方法的准确度和精密度较好,符合检测要求。运用*t*检验对两个仪器平台数据进行分析,结果显示:各化合物回收率的*P*为0.18,>0.05, RSD的*P*为0.17,>0.05;说明两个仪器平台间的准确度与精密度较为一致。

**表2:** 12种化合物在a-Ⅰ和柱b-Ⅱ上4个水平下的回收率和精密度(*n*=6)

Compound	Level^#^	Column a-Ⅰ			Column b-Ⅱ	
		Recovery/%	RSD/%		Recovery/%	RSD/%
Methanol	0.20	100.0	5.2		106.2	4.7
	0.80	98.6	1.2		101.4	1.3
	2.00	96.7	3.7		99.6	1.8
	3.00	100.7	2.3		98.9	1.3
Ethanol	0.20	109.1	1.6		107.3	3.9
	0.80	96.7	0.4		101.1	1.6
	2.00	92.2	2.6		100.3	2.3
	3.00	100.7	1.0		99.0	0.5
Isopropanol	0.20	99.5	3.2		106.1	1.5
	0.80	97.4	2.0		99.7	0.6
	2.00	97.0	1.0		101.2	1.2
	3.00	99.4	1.6		100.0	0.9
Acetone	0.20	111.6	1.2		107.1	0.8
	0.80	98.9	1.0		96.0	4.4
	2.00	97.2	1.3		99.7	1.9
	3.00	100.7	1.7		98.9	2.2
*N*-Propanol	0.20	106.3	5.8		110.8	1.4
	0.80	98.8	1.9		101.0	0.8
	2.00	95.2	0.9		97.9	4.4
	3.00	102.8	0.9		99.4	0.4
Isobutanol	0.20	105.6	7.4		111.2	0.5
	0.80	97.8	1.4		101.2	1.0
	2.00	96.2	2.1		98.1	3.3
	3.00	102.3	0.9		99.6	0.9
*N*-Butanol	0.20	105.4	1.2		107.9	3.3
	0.80	97.9	1.1		100.6	0.4
	2.00	93.6	0.7		101.1	2.1
	3.00	101.3	0.4		99.9	0.7
Benzene	0.50	102.8	1.7		98.9	1.9
	1.00	95.8	4.2		99.6	3.2
	5.00	100.1	1.2		98.5	1.2
	25.00	98.7	1.2		98.5	1.0
Toluene	0.50	98.7	2.4		105.1	0.8
	1.00	101.7	2.6		103.2	2.3
	5.00	96.6	1.9		97.8	1.3
	25.00	99.7	1.8		99.8	1.7
*p*-Xylene	0.50	100.7	2.5		100.2	2.0
	1.00	101.0	4.0		94.2	4.0
	5.00	97.5	1.2		97.2	1.8
	25.00	101.0	3.3		101.7	2.4
*m*-Xylene	0.50	96.6	2.0		95.8	1.2
	1.00	100.0	4.7		97.5	1.0
	5.00	99.7	0.5		98.8	1.1
	25.00	98.1	2.4		101.8	2.5
*o*-Xylene	0.50	100.2	3.0		100.9	2.7
	1.00	106.4	3.0		99.2	2.5
	5.00	95.2	1.2		96.5	1.1
	25.00	98.9	4.5		101.2	1.2

# The units for acetone and alcohols are g/L. The units for benzene compounds are mg/L.

为实现两个技术标准在同一个实验环境下的合并,本方法对色谱柱升温程序以及试剂纯度进行了适当的方法偏离。所用叔丁醇(99.8%)、邻二甲苯(98%)纯度低于标准要求(99.9%和99.5%,参考GB/T 42430-2023),总体实验结果表明,上述偏离对方法的核心技术参数所产生的影响并未超出技术标准的要求;同分析批次使用同一来源的叔丁醇作为内标,邻二甲苯的纯度经准确折算后,影响可有效降低。

### 2.2 乙醇的不确定度评定

根据《化学分析中不确定度的评估指南》及《测量不确定度评定与表示》,对两套设备平台上乙醇的测定结果进行不确定度评定,参照文献[18,19]的评定过程,所得各个分量的相对标准不确定度见表3。

**表3:** 两套设备平台及色谱柱体系上乙醇的相对标准不确定度分量

Item	Relative standard uncertainty components			
	Source	Symbol	Numeric values	
			Column a-Ⅰ	Column b-Ⅱ
A	determination repeatability	*u*_rel_(A)	3.59×10^-3^	5.03×10^-3^
B_1_	standard curve fitting	*u*_rel_(B_1_)	2.14×10^-2^	2.30×10^-2^
B_2_	standard solution	*u*_rel_(B_2_)	2.49×10^-3^	2.49×10^-3^
B_3_	pipette (50-250 μL)	*u*_rel_(V_100_)	4.08×10^-3^	4.08×10^-3^
B_3_	pipette (100-1000 μL)	*u*_rel_(V_500_)	2.04×10^-3^	2.04×10^-3^
B_4_	instrumental determination	*u*_rel_(GC)	5.00×10^-3^	5.00×10^-3^

计算得到两套设备平台及色谱柱体系上乙醇的合成相对标准不确定度分别为2.29×10^-2^(column a-Ⅰ)和2.46×10^-2^(column b-Ⅱ)。结果显示,不确定度主要来源于校准曲线,而非仪器平台。进一步分析,工作溶液配制是最显著的不确定度贡献者^[20,21]^,建议在日常检测中,通过购买低不确定度的成套系列浓度试剂来降低此影响;在本研究中,使用了外置活塞式移液器,相比使用传统移液器^[22⇓-24]^,由移液器带来的不确定度明显降低;因为血液属于黏性液体,使用合理的移液方案能有效降低不确定度。此外,对于挥发性物质应该选择合适的容器和密封方式,远离热源,防止样品中的物质逸散损失以及外环境对样品的污染。

### 2.3 实际样品分析

#### 2.3.1 能力验证样品分析

对实验室接收的2个能力验证样品用所建立的方法进行测试,以双柱定性、单柱定量。每个仪器平台每份样品平行取两份,分别各测试一次,以两个测试值的平均值作为含量测定结果(*C*, *n*=2),两个测试值之间的差值除以这两个数值的平均值作为双样相对相差(RD)。

经分析与计算,在柱a-Ⅰ上,样品1的乙醇含量测定结果*C*_PT-sample1-a-Ⅰ_=1.20 g/L, RD_PT-sample1-a-Ⅰ_=2.6%(不超过标准中所规定的10%,含量测定结果有效);在柱b-Ⅱ上,该样品的乙醇含量测定结果*C*_PT-sample1-b-Ⅱ_=1.20 g/L, RD_PT-sample1-b-Ⅱ_=2.5%。对于样品2, *C*_PT-sample2-a-Ⅰ_=0.78 g/L, RD_PT-sample2-a-Ⅰ_=2.0%, *C*_PT-sample2-b-Ⅱ_=0.79 g/L, RD_PT-sample2-b-Ⅱ_=2.2%。

经与当批次能力验证数据集比较,4个定量数据结果的*z*比分数(*z*-score)分别为0.51、0.45、0.51、0.67,均符合中国合格评定国家认可委员会当批次能力验证计划样品测定的相关要求。取置信概率95%(包含因子*k*=2),能力验证样品1在柱a-Ⅰ和柱b-Ⅱ上的含量测定结果分别为(1.20±0.06) g/L和(1.20±0.07) g/L,样品2在柱a-Ⅰ和柱b-Ⅱ上的含量测定结果分别为(0.78±0.04) g/L和(0.79±0.05) g/L;在不同仪器平台上,同一样品的RD均未超过10%;样品1的扩展不确定度为0.06和0.07,样品2的扩展不确定度为0.04和0.05,不确定度亦较为接近,说明所建立方法能适用于不同仪器平台,定量结果具有较高的一致性。

#### 2.3.2 中毒案例样品分析

案例一:实验室于2023年9月收到疑似挥发性溶剂中毒患者血液一份,用所建立方法进行分析,以双柱定性、单柱定量;疑似挥发性溶剂中毒患者血液在柱a-Ⅰ、a-Ⅱ、b-Ⅰ、b-Ⅱ上的色谱图见图2。经分析,平台a与平台b均检出甲醇、对二甲苯、间二甲苯、邻二甲苯(图2),此外,经标准物质比对,还检出了二氯甲烷、乙酸乙酯。对甲醇、对二甲苯、间二甲苯和邻二甲苯进行定量分析,在柱a-Ⅰ上甲醇、对二甲苯、间二甲苯和邻二甲苯的含量测定结果分别为0.30 g/L、20.1 mg/L、87.1 mg/L、39.8 mg/L, RD分别为0.34%、3.5%、0.15%、3.6%;在柱b-Ⅱ上,甲醇、对二甲苯、间二甲苯和邻二甲苯的含量测定结果分别为0.32 g/L、18.90 mg/L、86.5 mg/L、40.0 mg/L, RD分别为4.5%、2.4%、1.1%、0.17%。其中间二甲苯的首次定量结果超出线性范围,参照GB/T 42430-2023要求对样本稀释10倍二次定量。该分析结果为临床急救提供了重要指标。

**图2:**
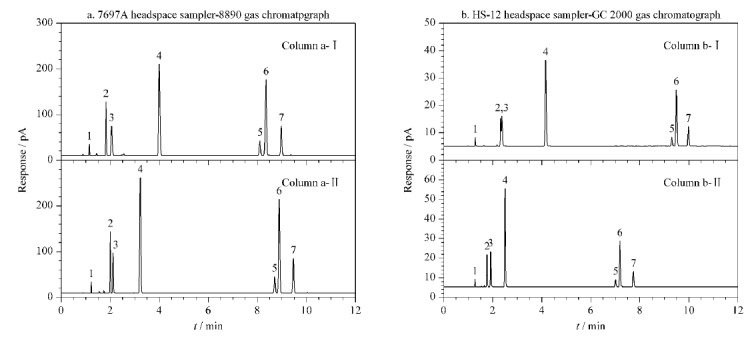
疑似挥发性溶剂中毒患者血液在柱a-Ⅰ、a-Ⅱ、b-Ⅰ、b-Ⅱ上的色谱图

案例二:实验室于2023年7月收到疑似假酒中毒患者血液和尿液各一份,按照1.3节处理,处理后的血液、尿液均用所建立的方法进行测试,以双柱定性、单柱定量。检测结果见表4,案例二甲醇所检出浓度接近文献报道的急性致死浓度(0.90 g/L)^[25,26]^,中毒特征与文献相仿。

**表4:** 案例二在柱a-Ⅰ和b-Ⅱ上的测定结果(*n*=2)

Sample	Column a-Ⅰ				Column b-Ⅱ		
	Compound	Assay result/(g/L)	RD/%		Compound	Assay result/(g/L)	RD/%
Blood	methanol	0.83	1.1		methanol	0.87	3.1
	ethanol	0.48	0.40		ethanol	0.52	2.8
	acetone	+	+		acetone	+	+
Urine	methanol	0.78	0.58		methanol	0.83	2.8
	ethanol	0.20	2.1		ethanol	0.24	0.16
	acetone	-	-		acetone	-	-

RD: standing for relative difference, calculated by dividing the difference between the outcomes of two tests by their average. +: detected but lower than the LOQ; -: not detected.

在案例一中,不仅展示了一个方法能同时适应两项不同技术标准,体现了方法的高效性,同时也展示了方法能实现对标准所列目标物以外化合物的检测。两个案例应用展现了所建立方法在实践中的适应性与可拓展性。在案例二中,成功分析到患者体内甲醇、乙醇含量,并监测到微量丙酮存在,提示患者酮中毒可能性,为临床急救提供了有效数据,同时也说明所建立方法符合GB/T 42430-2023所述可拓展应用于其他人体体液分析。

## 3 结论

本研究建立了一套能同时符合GB/T 42430-2023与GA/T 204-2019标准要求的挥发性物质分析技术方法。方法可在两套设备平台、不同色谱条件下得到相似的结果。经比较,所使用的国产顶空进样-气相色谱平台与进口设备平台在方法学结果上未见显著性差异,说明国产仪器在本方法的应用中具备替代能力。经验证,J&W DB-BAC2 UI色谱柱(柱a-Ⅱ, 30 m×0.32 mm×1.2 μm)与Rtx-BAC-PLUS1色谱柱(柱b-Ⅰ, 30 m×0.32 mm×1.8 μm)分别在苯与异丁醇的分离、苯与正丁醇的分离上难以同时实现,提示在实践中应更充分地考察多目标物分析时的定性有效性;也体现了在缺乏质谱等辅助定性检测手段的情况下,使用不同性质色谱柱进行分析,具有减少不同化合物在一种性质的色谱柱上可能因保留时间相同引起误判的作用。以J&W DB-BAC1 UI色谱柱(柱a-Ⅰ, 30 m×0.32 mm×1.8 μm)或Rtx-BAC-PLUS 2色谱柱(柱b-Ⅱ, 30 m×0.32 mm×0.6 μm)为基础建立的定量分析相关参数均符合各标准要求。通过实际样品分析,说明了方法的有效性以及在面对多场景应用时的适应性和可拓展性,可用于含道路交通事故涉酒司法鉴定在内的多种挥发性物质分析实践场景。
